# Probing sources of strontium exposure in pregnant individuals living near unconventional oil and gas wells using urinary ^87^Sr/^86^Sr isotope ratios

**DOI:** 10.1038/s41370-025-00784-0

**Published:** 2025-06-19

**Authors:** Karel Houessionon, Bruna Saar de Almeida, David Widory, Michèle Bouchard, Vikki Ho, Coreen Daley, Élyse Caron-Beaudoin, Delphine Bosson-Rieutort, Marc-André Verner

**Affiliations:** 1 Department of Occupational and Environmental Health, School of Public Health, Université de Montréal, Montréal, QC Canada; 2 Centre de Recherche en Santé Publique, Université de Montréal and CIUSSS du Centre-Sud-de-l’Île-de-Montréal, Montreal, QC Canada; 3 GEOTOP, Université de Québec à Montréal, Montréal, QC Canada; 4 Chaire d’analyse et de gestion des risques toxicologiques, Université de Montréal, Montréal, QC Canada; 5 Department of Social and Preventive Medicine, School of Public Health, Université de Montréal, Montréal, QC Canada; 6 Health Innovation and Evaluation Hub, Université de Montréal Hospital Research Centre (CRCHUM), Montréal, QC Canada; 7 Department of Physical and Environmental Sciences, University of Toronto Scarborough, Toronto, ON Canada; 8 Department of Health and Society, University of Toronto Scarborough, Toronto, ON Canada; 9 Department of Health Policy, Management and Evaluation, School of Public Health, Université de Montréal, Montréal, QC Canada

**Keywords:** ^87^Sr/^86^Sr, Strontium, Exposure, Hydraulic fracturing, Pregnant individuals

## Abstract

**Background:**

In the Exposures in the Peace River Valley (EXPERIVA) study, pregnant individuals living in a region of natural gas exploitation had higher biological concentrations of certain trace elements, including strontium (Sr), than the general population. However, sources remained unidentified.

**Objectives:**

To measure urinary ^87^Sr/^86^Sr isotope ratio in EXPERIVA participants, assess its reliability, and explore how its variance fluctuates based on Sr concentrations in biological (urine, hair, nails) and environmental (tap water) samples, as well as the density/proximity of unconventional oil and gas wells around participants’ residence.

**Methods:**

Participants provided urine daily over seven consecutive days. We measured ^87^Sr/^86^Sr in each urine sample from 7 participants and in pooled daily samples for all 75 participants. We used serial measurements to determine the intraclass correlation coefficient (ICC). We calculated the density/proximity of unconventional oil and gas wells around participants’ homes using inverse distance weighting (IDW). We assessed the variance of urinary ^87^Sr/^86^Sr based on Sr concentrations in biological/environmental samples and IDW through visual inspection and Levene’s test. We also performed unsupervised clustering to explore whether certain characteristics of the participants may be associated with a specific ^87^Sr/^86^Sr signature.

**Results:**

Urinary ^87^Sr/^86^Sr ranged from 0.70798 to 0.71437. The ICC was 0.797 (95% CI: 0.574–0.953), indicating moderate to excellent reliability. Increasing Sr concentrations in hair were marginally associated with a decrease in urinary ^87^Sr/^86^Sr variance (*p* = 0.066). A similar but less consistent association was observed with increasing IDW. We observed no association between Sr concentrations in water and variance in urinary ^87^Sr/^86^Sr. No clear pattern was found using unsupervised clustering.

**Impact:**

To our knowledge, this study is the first to explore the use of urinary ^87^Sr/^86^Sr isotope ratios to investigate sources of Sr exposure. Results are consistent with the hypothesis that a predominant source contributes to Sr exposure in most exposed EXPERIVA participants, but the contribution of unconventional oil and gas wells around participants’ residences remains unclear. Findings should be considered as exploratory given the many limitations of this study. Our effort will hopefully benefit future studies aimed at identifying the sources of exposure in human populations.

## Introduction

Northeastern British Columbia, and more specifically the Peace River Valley region, is known for its important natural gas production, namely through hydraulic fracturing [1–5]. During hydraulic fracturing operations, contaminants naturally present in the rocks can be released into the air, water, and soil through accidental spills and improper treatment and storage of flowback water [6, 7]. Studies in the United States have reported high concentrations of certain trace elements like sodium (Na), barium (Ba), and strontium (Sr) in water samples from hydraulic fracturing operations [8]. In Northeastern British Columbia, at least 2,329 wells have had a reported leakage event over 21,525 tested wells. The actual number of leaking wells is probably higher due to underreporting [9].

In the Exposures in the Peace River Valley (EXPERIVA) study, pregnant individuals had higher biological levels of certain trace elements than reference populations, including vanadium (V), gallium (Ga), cobalt (Co), Ba, and Sr. For example, the median urinary Sr concentration in participants was 195 µg/g creatinine, almost twice that in women from the 9^th^ cycle of the US National Health and Nutrition Examination Survey (NHANES) [1]. Whether Sr exposure in EXPERIVA participants represents health risks is uncertain. The Human Biomonitoring Health-Based Guidance Value Dashboard [10] contains no value for Sr, preventing direct comparisons to assess risk associated with measured levels. Some epidemiological studies with similar urinary Sr levels during pregnancy or infancy have reported associations with outcomes like fetal growth [11, 12] and neurodevelopment [13, 14], but results were inconsistent. Previous work by our team using the rat fetal testis assay showed that Sr can increase testosterone secretion alone or in mixtures [15]. Overall, the limited evidence suggests early-life exposure to Sr may be associated with fetal/child development, warranting efforts to identify sources and prevent exposure. A previous analysis within the EXPERIVA study revealed a correlation between the density/proximity of gas wells and Sr concentrations in tap water [16]. However, the sources of Sr exposure are diverse and multifaceted, encompassing natural origins such as rocks, soils, and groundwater, as well as anthropogenic contributions from industrial activities and agriculture [17]. The specific sources of exposure to Sr in EXPERIVA participants remain elusive.

Identifying the sources of exposure is critical to establish exposure mitigation strategies. A potential approach to determine the specific sources of exposure is the use of isotope ratios. Among the trace elements that were measured at higher concentrations in EXPERIVA compared to reference populations, Sr is particularly amenable to isotopic tracing [18]. Based on the specificity of the distribution of ^87^Sr and ^86^Sr isotopes, reflecting the origin, age and composition of geological terrains, ^87^Sr/^86^Sr ratios have been recently used as a tool for tracking human mobility and identifying sources of Sr [18–22]. Indeed, ^87^Sr/^86^Sr in hair has been shown to be influenced by the subject’s living environment and thus may fluctuate if the subject relocates, as observed in the study of Sr isotope ratios in the drinking water and hair of women who emigrated from France to Eastern Canada [18]. A study in Mexico also showed a statistically significant association between ^87^Sr/^86^Sr in tap water and human hair [23]. With regards to hydraulic fracturing, researchers in China used ^87^Sr/^86^Sr to identify and apportion the contribution of hydraulic fracturing flowback fluid in local groundwater [7]. Together, these studies suggest that environmental and biological ^87^Sr/^86^Sr isotope ratio measurements show potential in investigating sources of Sr.

In this study, we aimed to measure the ^87^Sr/^86^Sr isotope ratio in urine from EXPERIVA participants, assess its reliability to distinguish between individuals using repeated measurements in a subset of participants, and explore how its variance fluctuates based on Sr concentrations in biological (urine, hair, nails) and environmental (tap water) samples, as well as the density/proximity of unconventional oil and gas wells around participants’ residence. Unlike previous EXPERIVA studies focused on trace element concentrations, the use of ^87^Sr/^86^Sr isotope ratios offers novel, source-specific insights into Sr exposure and its potential environmental origins.

## Materials and methods

### Recruitment and study population

The study population consisted of pregnant individuals living in the Peace River Valley (Northeastern British Columbia), and has been described elsewhere [5]. Briefly, pregnant individuals aged ≥18, living in the Peace River Valley area, available during the data collection period and having given their consent to participate in the study were included in the present study. A total of 85 participants were recruited between May and September 2019 and completed questionnaires, and biological (i.e., urine, hair, nails) and tap water samples collection. Out of these, 75 had sufficient remaining urine volume for ^87^Sr/^86^Sr isotope ratio measurements. A questionnaire was administered by the research team, and collected information on pregnancy, anthropometric measurements, place of residence, income, occupation, habits, diet, drinking water and perceived health and quality of living environment. The EXPERIVA study was approved by the Northern Health Research Review Committee and the Clinical Research Ethics Committee of the Université de Montréal (#CERC-18-003-P). Informed consent was obtained from all subjects.

### Sampling

As previously described [5], EXPERIVA participants were asked to collect urine samples in 50 mL polypropylene tubes once a day, between dinner and bedtime, for seven consecutive days. Urine samples were first stored in the participants’ respective freezers at a temperature of −20 °C. These samples were then collected by the research team and transported, with cold accumulators, to the laboratory in Montreal for analysis. A hair sample was collected from participants, from scalp to tip. Samples were stored in plastic bags at room temperature. The first 2 cm closest to the scalp were used for analysis. Based on a hair growth rate of 1 cm/month [24], the first 2 cm are assumed to reflect the body burden during the two months prior to collection. Nail samples were collected by cutting the distal free edge of each toenail for participants who agreed to provide nail samples and who did not have nail polish or were willing to remove polish. Samples were stored in a 1.5 mL centrifuge tube at room temperature until analysis. Given that the distal free edge of the nail is isolated from the body’s metabolic activities, concentrations measured in this section reflect the body burden months prior to collection. Tap water samples were taken from the kitchen tap in the participants’ home in duplicate, using 15 mL polypropylene tubes. The first sample was taken directly when the water began to flow, and the second after 5 min of flow. Samples were transported to the laboratory on ice and stored at −20 °C until analysis.

### Trace element analyses

Trace element analyses were carried out at the Université de Montréal “Xenobiotics and Nanoparticles” platform. Sr concentrations, alongside 20 other trace elements, were measured in urine, nail, hair and tap water samples using inductively coupled mass spectrometry (ICP-MS) 7700x (Agilent, Mississauga, Canada) in a clean room for trace elements (ISO 3 standards 146442-1). The methods and concentrations are detailed in previous publications by the research team [2, 16].

### Measurement of urinary ^87^Sr/^86^Sr isotope ratios

We measured ^87^Sr/^86^Sr isotope ratios in serial urine samples for 7 participants (to evaluate reliability), and in pooled daily urine samples from all participants with sufficient urine volume (n = 75). ^87^Sr/^86^Sr isotope ratios were determined, after Sr purification, by thermal ionization mass spectrometry (TI-MS) at the GEOTOP laboratory (Université du Québec à Montréal). A urine volume of 3 mL was mixed with 5 mL of Mill-Q H_2_O, 18.2 MΩ cm, in a 15 mL Teflon tube, and purified by cation exchange chromatography using a AG50-X8 resin (3–5 mL of 100–200 mesh resin in a 1 N HCl solution) in Bio-Rad columns (10 mL). The resin was cleaned by passing 5 mL of 6 N HCl (three times), followed by 5 mL of 1 N HCl (three times). Samples were manually shaken (into Teflon tubes) and poured into the columns twice (7.5 mL each). Organics were rinsed out using 5 mL of 1 N HCl (three times), and cations were recovered using 5 mL of 6 N HCl (twice). Samples were subsequently heated on a hot plate at 85 °C for 24 h until dryness. 1 mL of 8 N HNO_3_ was then added. Samples were placed in an ultrasonic bath for 10 min and centrifuged for 10 min before loading into the separation columns. Sr was extracted by cation exchange chromatography using 0.25 mL of Eichrom Sr-spec resin in a 1 mL Bio-RadTM column. The resin was washed using 2 mL of Mill-Q H_2_O (one time), and 2 mL of 8 N HNO_3_ (three times). Samples were loaded (0.25 mL of sample in four times), rinsed (using 0.5 mL of 8 N HNO_3_ for six times) and recovered (using 0.25 mL of 0.05 N HNO_3_ for five times). At the end of the procedure, samples were dried at 85 °C for 24 h. Samples were analyzed by TI-MS after adding 1 µL of 16 N HNO_3_ and loaded on the Re-filaments using 0.8 µL of tantalum oxide activator.

### Data analysis

The density/proximity of unconventional oil and gas wells around participants’ home was calculated using British Columbia Energy Regulator (BCER) gas exploitation databases, QGIS and R software as described previously [25]. The BCER data contains information on around 35,504 oil and gas wells in British Columbia. Unconventional oil and gas wells were identified by selecting wells with an indicator of directional drilling or if they had a count of fracking event. The density/proximity of unconventional oil and gas wells were calculated using the inverse distance weighting (IDW) approach within a buffer of 5 km around participants’ home and without buffer. We assumed that wells may contribute to environmental contamination starting at the initial drilling stage and onwards (including after operations have ceased). Therefore, all wells with a spud date prior to delivery were considered for IDW calculations. The following equation was used:

1 $${{IDW}}_{x}={\sum }_{i=1}^{n}\left(\frac{1}{{d}^{2}}\right)$$

where “x” is the buffer size (km) around the participant’s residence; “i” is a given unconventional oil and gas well within the buffer; “d^2^” is the distance squared (km) between that unconventional well and the participant’s residence; and “n” is the total number of unconventional wells within the buffer.

Several studies have shown that a single biological sample may not adequately represent average exposure for certain chemicals [26–28]. To assess the reliability of urinary ^87^Sr/^86^Sr isotope ratios in spot urine samples, we calculated an intraclass correlation coefficient (ICC) based on repeated ^87^Sr/^86^Sr measurements from 7 participants. ICCs compares the between-subject variance to the sum of within-subject and between-subject variance, with values ranging from 0 to 1. In the current study, a high ICC value would mean that a substantial proportion of the total variance in urinary ^87^Sr/^86^Sr is attributable to differences between individuals, indicating that urinary measurements can be used to reliably distinguish individuals. Typically, reliability is considered poor if ICCs are below 0.5, moderate between 0.5 and 0.75, good between 0.75 and 0.9, and excellent above 0.9 [29]. We calculated an ICC and 95% confidence intervals using the “irr” package under R software and “one-way random effects model”, “single rater”, “consistency” method, as recommended in the scientific literature [29].

When working with isotope ratios, we assume that large variance indicates multiple sources of exposure with distinct isotope fingerprints, whereas minimal variance indicates a predominant source with a specific isotope signature. In the context of this study, we were interested in determining whether the participants with higher Sr exposure or with more unconventional oil and gas wells around their residence have lower variance in urinary ^87^Sr/^86^Sr, which would suggest a predominant source of Sr. We used two approaches to assess changes in the variance of urinary ^87^Sr/^86^Sr with increasing Sr concentration in urine, nails, hair and tap water (average of both water sample concentrations), on one hand, and with increasing IDW metrics on the other. We first visually evaluated patterns of ^87^Sr/^86^Sr dispersion with increasing Sr concentrations or IDW using scatter plots. Our second approach was to stratify participants into quartiles based on Sr concentrations or IDW, and to compare variance in urinary ^87^Sr/^86^Sr across quartiles using the Levene’s test.

Additionally, we conducted unsupervised clustering to classify the participants according to their characteristics (i.e., measured exposures, data collected by questionnaire), excluding urinary ^87^Sr/^86^Sr. The objective was to evaluate if certain clusters had lower variance in ^87^Sr/^86^Sr, which would indicate a predominant source of exposure to Sr related to cluster-specific characteristics. To achieve this, we applied a hierarchical ascendant classification using the ward.D method on a binary dissimilarity matrix. A Levene’s test was used to evaluate whether the variance in urinary ^87^Sr/^86^Sr differed across clusters.

The statistical analyses and graphs in this article were produced using R software version 4.3.2.

## Results

### Characteristics and lifestyle habits of the study population

Table 1 shows the sociodemographic and lifestyle characteristics of participants included in this study. The average age was 34 years, ranging from 23 to 45 years. Almost half of participants had a technical diploma, university degree or vocational certificate. More than half reported an annual household income of more than 100,000 Canadian dollars. A description of the participants’ lifestyle habits revealed that most did not engage in any occupational activities that would have particularly exposed them to Sr. More than one-third of the participants consumed wild meat and wild berries, and less than half consumed fruits and vegetables from local gardens. In terms of domestic water use, over half of the participants consumed tap water, whether filtered or not, while the remainder preferred bottled water for drinking. However, tap water was used for cooking by almost all participants. Approximately two-thirds of participants used water supplied by the municipality as their primary source of water for domestic use (Table 1).

**Table 1:** Characteristics of EXPERIVA participants.

Characteristics	Groups	*N* (%)
**Age (years)**	≤29	12 (16)
	30-4	30 (40)
	35–39	27 (36)
	>40	6 (8)
**Years lived at current residence**	<1	21 (28)
	1–2	17 (23)
	2–5	21 (28)
	>5	16 (21)
**Highest degree**	<10^th^ grade	2 (3)
	11^th^ grade	8 (11)
	12^th^ grade	10 (13)
	Technical degree/certificate	36 (48)
	Bachelor	15 (20)
	Master	4 (5)
**Household income/year (CAD)**	0–19,999	1 (1)
	20,000–49,999	7 (9)
	50,000–99,999	24 (32)
	>100,000	41 (55)
	Do not know	2 (3)
**Working in industry**	Yes	7 (9)
	No	68 (91)
**Wild meat consumption**	Yes	29 (39)
	No	46 (61)
**Wild berries consumption**	Yes	25 (33)
	No	50 (67)
**Garden fruits and vegetables consumption**	Yes	35 (47)
	No	40 (53)
**Consumption of caught fish**	Yes	10 (13)
	No	65 (87)
**Drinking water**	Tap water	24 (32)
	Filtered tap water	19 (25)
	Bottled water	32 (43)
**Cooking water**	Tap water	67 (89)
	Filtered tap water	3 (4)
	Bottled water	5 (7)
**Household water source**	Municipality	42 (56)
	Private wells	5 (7)
	Cistern	22 (29)
	Surface water	1 (1)
	Other	5 (7)

### Urinary ^87^Sr/^86^Sr and exposure metrics

**Fig. 1:**
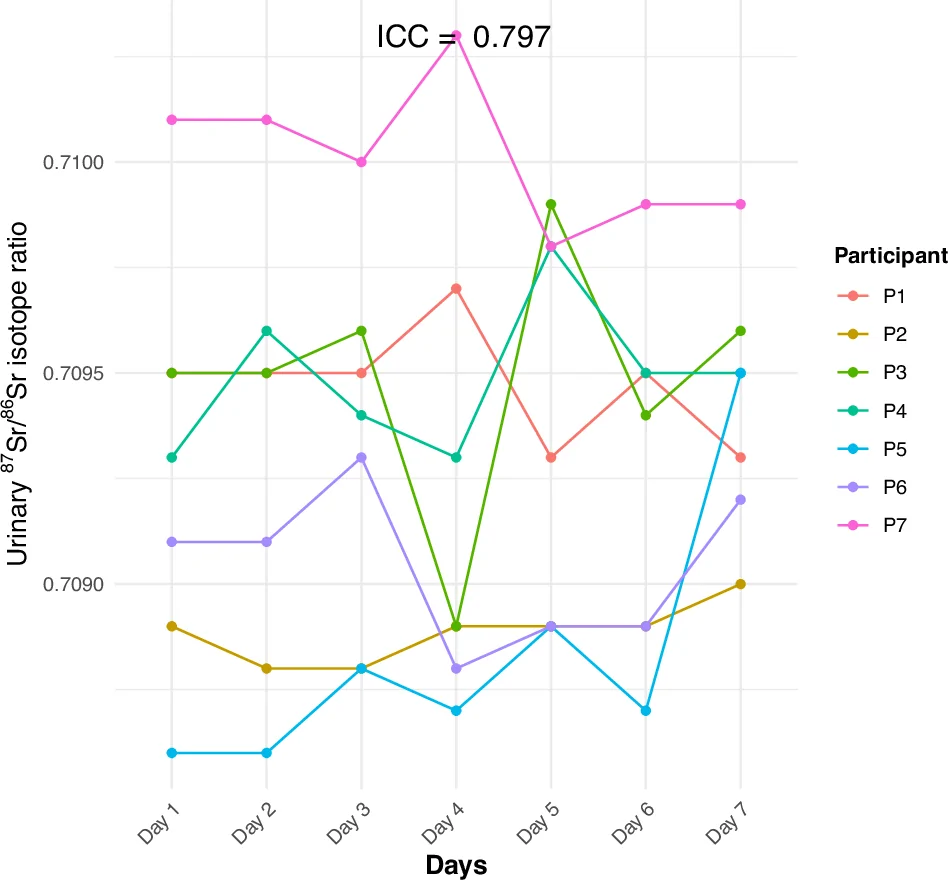
Urinary ^87^Sr/^86^Sr isotope ratios in serial urine samples (7 participants). Each color represents a study participant (P1 to P7). The intraclass correlation coefficient (ICC) was calculated using repeated measurements from these 7 participants.

Urinary ^87^Sr/^86^Sr in pooled samples varied between 0.70798 and 0.71437. The obtained ICC value was 0.797 (95% confidence intervals: 0.574–0.953), indicating a moderate to excellent reliability of the measured ratios to distinguish between individuals (Fig. 1).

**Fig. 2:**
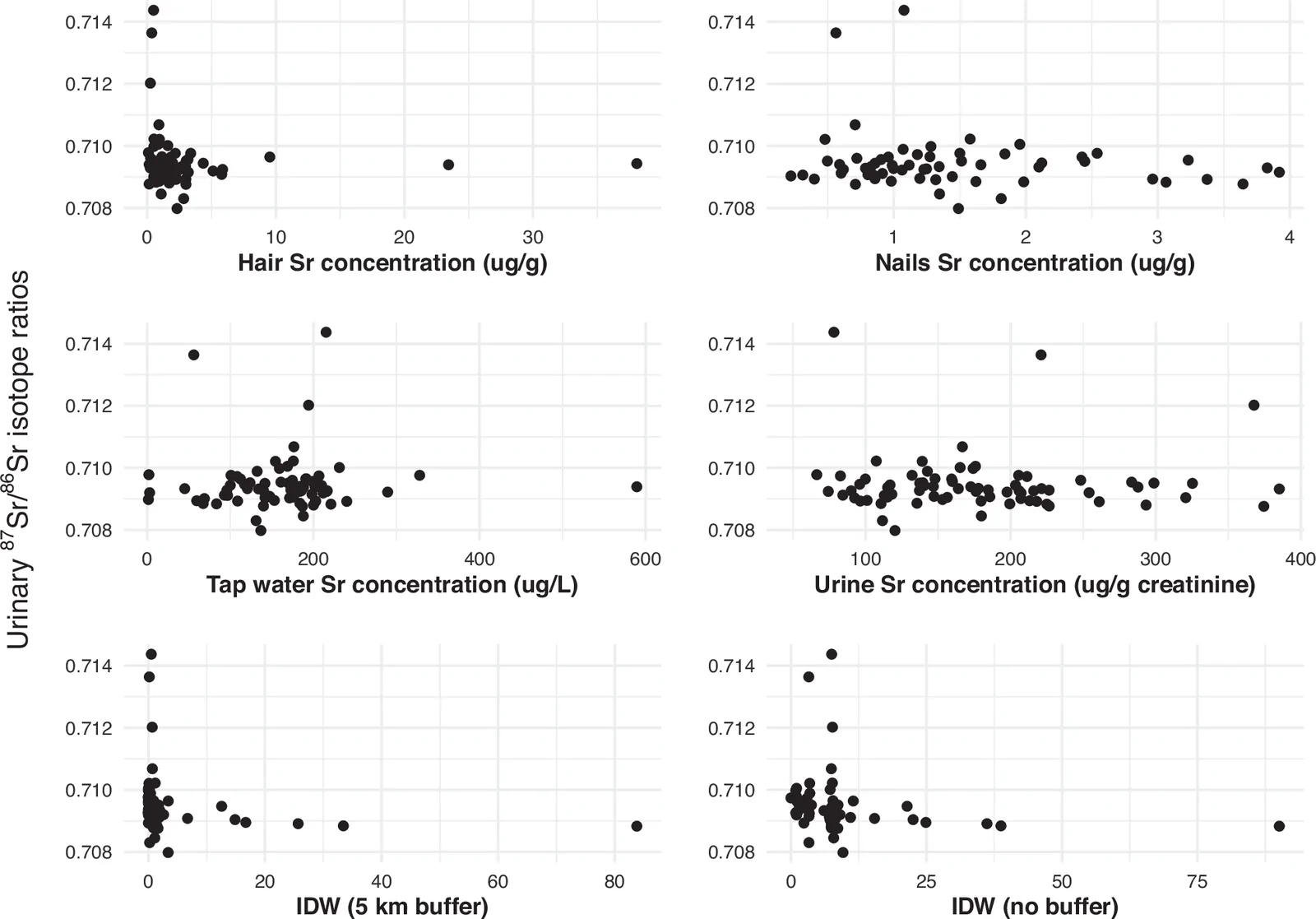
Distribution of urinary ^87^Sr/^86^Sr isotope ratios with increasing measured Sr concentrations (in hair, nails, urine and tap water) or IDW metrics.

**Fig. 3:**
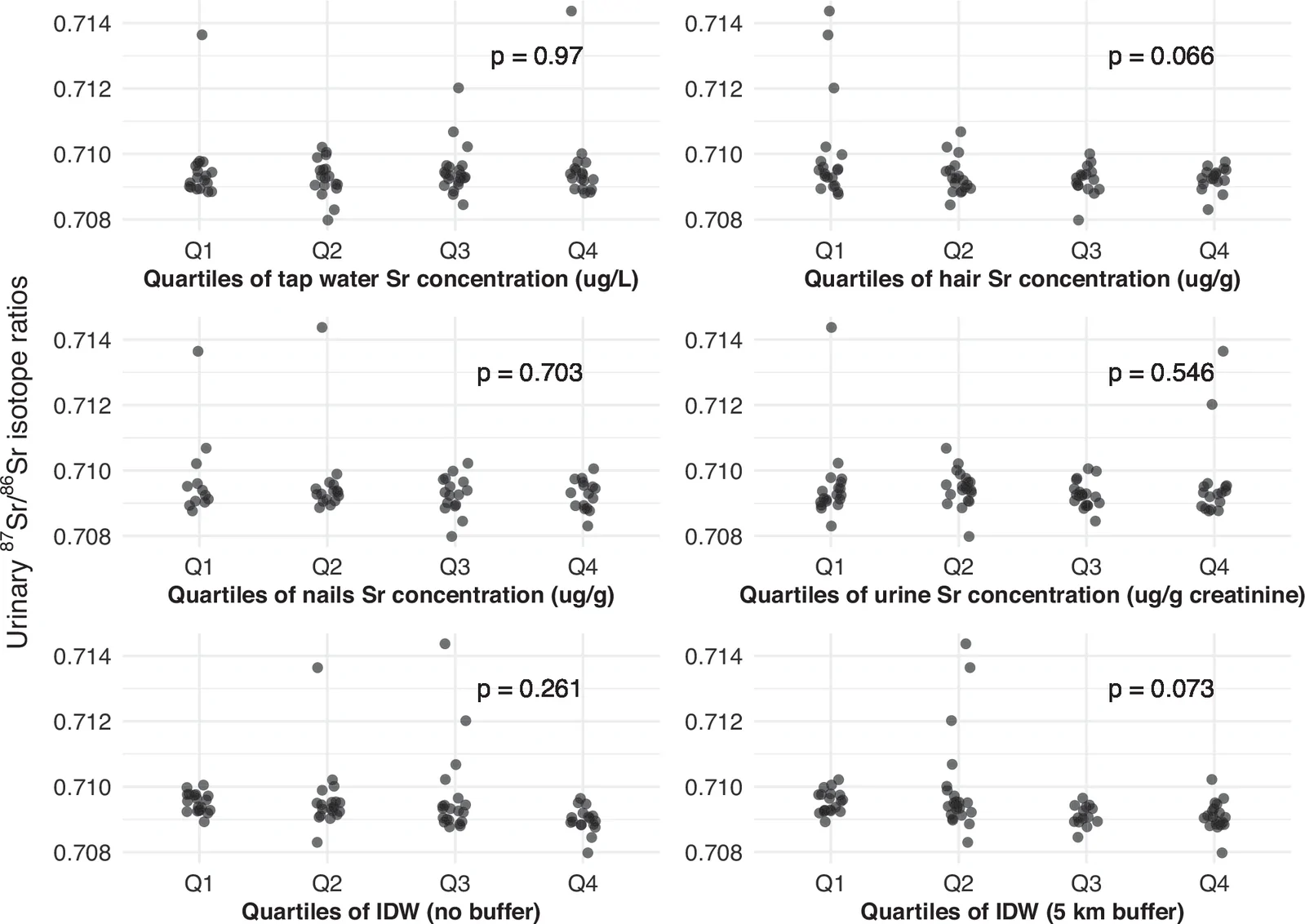
Distribution of urinary ^87^Sr/^86^Sr isotope ratios in quartiles of measured Sr concentrations (in hair, nails, urine and tap water) or IDW metrics. Levene’s tests were used to evaluate differences in variance across groups (*p* values are indicated in the plots).

The variability of urinary ^87^Sr/^86^Sr with increasing exposure metrics was visually inspected using the scatter plots presented in Fig. 2. We observed a pattern of decreasing ^87^Sr/^86^Sr variability as hair Sr concentrations increased, with ^87^Sr/^86^Sr values converging towards 0.7090. The same trend was observed with increasing IDW (5 km) and IDW (no buffer). The downward trend in the variability of urinary ^87^Sr/^86^Sr isotope ratios was less visible with increasing Sr concentrations in nail and urine samples. No pattern was observed with increasing Sr concentrations in tap water samples. When urinary ^87^Sr/^86^Sr variance was evaluated across quartiles of biological Sr concentrations, tap water concentrations or IDW, all *p* values from Levene’s tests were above 0.05 (Fig. 3), suggesting equal ^87^Sr/^86^Sr variance in the different quartiles. Only with hair Sr concentrations and IDW (5 km) were the *p* values between 0.05 and 0.10 (Fig. 3). A decrease in urinary ^87^Sr/^86^Sr variance was observed in the highest quartiles of hair Sr concentrations (consistent with the visual inspection of the scatter plots), but the pattern was not as clear with quartiles of IDW (5 km) where only the second quartile displayed increased ^87^Sr/^86^Sr variance compared to the other quartiles.

### Clustering analysis

**Fig. 4:**
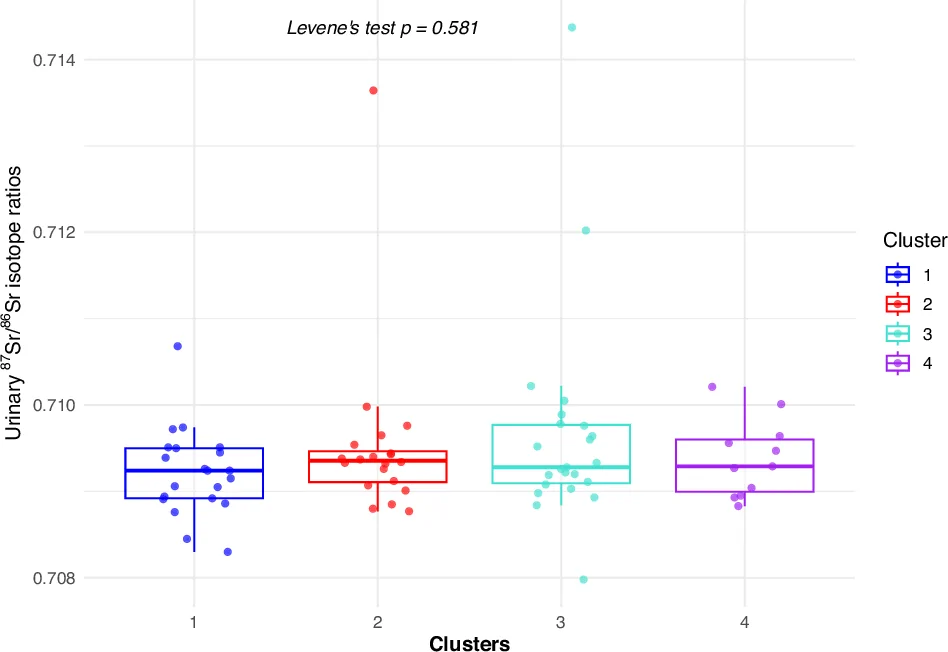
Distribution of urinary ^87^Sr/^86^Sr isotope ratios within clusters. Levene’s test was used to evaluate differences in variance across clusters (the *p* value is indicated in the plot). The different clusters are presented in the text, and in heatmaps provided in Supplementary Information (Figs. S1, S2).

Unsupervised clustering analysis resulted in 4 distinct clusters. Median values for exposure metrics and lifestyle characteristics are depicted in Figs. S1, S2. Cluster 1 included participants (*n *= 21) with higher hair Sr concentrations, lower urinary ^87^Sr/^86^Sr, higher garden fruits/vegetable and caught fish intake, and higher use of tap water for cooking compared to participants in other clusters. Cluster 2 participants (*n* = 20) had lower tap water Sr concentrations, higher median urinary Sr concentrations, higher urinary ^87^Sr/^86^Sr, lower wild meat and berries intake, and mostly got their tap water from the municipality. Cluster 3 participants (*n* = 23) mostly differed from other clusters in terms of lower urinary Sr concentrations. Cluster 4 participants (*n* = 11) had higher tap water, hair and nail Sr concentrations, and lower IDW values. Figure 4 presents the distribution of urinary ^87^Sr/^86^Sr within the 4 clusters. Visual inspection and Levene’s test (*p* = 0.581) suggested that the variability in urinary ^87^Sr/^86^Sr was similar across the four clusters.

## Discussion

The present study aimed to evaluate urinary ^87^Sr/^86^Sr isotope ratio as a marker of Sr sources, and to explore whether variance in urinary ^87^Sr/^86^Sr changes with increasing Sr exposure or density/proximity of unconventional oil and gas wells around EXPERIVA participants’ residence. Visual assessments suggested that increasing hair Sr concentrations and, to a lesser extent, well density/proximity (IDW 5 km) were associated with a decrease in urinary ^87^Sr/^86^Sr variance, with values converging around 0.7090. A decrease in the variability of urinary ^87^Sr/^86^Sr suggests that Sr in samples originates from a single predominant source. Although Levene’s test *p*-values for IDW and hair Sr concentrations were between 0.05 and 0.10, suggesting they were not statistically significant at the conventional 0.05 threshold, they may indicate a trend toward reduced variance in urinary ^87^Sr/^86^Sr at higher exposures. This trend, while not definitive, may indicate that a single exposure source dominates at high exposure, and should be further evaluated in a larger study. No clear pattern was observed in terms of urinary ^87^Sr/^86^Sr variance in clusters of participants generated using unsupervised clustering.

In the present study, ^87^Sr/^86^Sr in pooled urine samples from participants ranged from 0.70798 to 0.71437, with a converging tendency towards 0.7090 at both higher IDW and hair Sr concentrations. This value is relatively close to the ^87^Sr/^86^Sr range of 0.7092 and 0.7102 reported by Liseroudi et al. [30] in fracture and cavity-filling anhydrite samples (minerals) in the local Montney geological formation in the Peace River Valley. This supports the hypothesis that water, which partly inherits its isotope signature from the interaction between groundwater and the local geology, may contribute to Sr exposure in participants. Another study showed that seawater from the Lower Triassic Montney formation yielded more radiogenic ^87^Sr/^86^Sr, ranging from 0.7108 to 0.7128 [31]. To our knowledge, ^87^Sr/^86^Sr has yet to be determined in flowback water from hydraulic fracturing operations in the Peace River Valley. Such measurements would be instrumental in more robustly determining whether flowback water leaks/disposal contributes to human exposure to Sr in the region.

Sr concentrations in biological samples reflect exposure from different sources and routes. According to Health Canada, Sr exposure occurs mainly through the consumption of contaminated water and/or food, depending on the levels of contamination of the agricultural soils where food was grown [32]. Sr concentrations in drinking water vary depending on water-rock interaction and anthropogenic activities. In a previous analysis using EXPERIVA data, Gasparyan et al. found a positive association between IDW (density/proximity) values and Sr concentrations in tap water samples [16]. However, our results did not indicate an association between tap water Sr concentrations and variance in urinary ^87^Sr/^86^Sr. This is in line with the absence of correlation between urinary Sr concentrations and tap water Sr concentrations previously reported in EXPERIVA participants [16]. Conversely, tap water Sr concentrations showed relatively weak but significant correlations with Sr concentrations in hair and nails. Subsequent studies should consider measuring ^87^Sr/^86^Sr in hair and nails to more thoroughly assess associations with gas well density/proximity.

Strengths of this exploratory study include the collection of repeated urine samples from study participants. Pooling daily urine samples allowed us to assess the average isotope ratio and reduced the impact of day-to-day variations (see individual measurements in Fig. 1). Another strength is the measurement of ^87^Sr/^86^Sr in repeated urine samples from 7 participants. While we recognize that a sample of 7 participants is limited, we were able to calculate the first ICC for ^87^Sr/^86^Sr in urine which suggested that urinary measurements provide a moderate to excellent reliability in distinguishing between individuals. On the other hand, our study has many limitations. We only measured isotope ratios in urine samples; additional measurements in hair and nail samples could have provided longer-term isotope profiles. Also, the lack of isotope characteristics from food (e.g., wild and/or locally grown produce) and environmental samples (e.g., hydraulic fracturing flowback water, soil surrounding wells) substantially reduced our ability to determine the environmental sources of Sr exposure for the participants. Our sample size was relatively small, which negatively impacted statistical power in our analyses and prevented us from performing stratified analyses (e.g., based on tap water source). Finally, given the small sample size and exclusion of participants with insufficient remaining urine volume, the generalizability of our findings to the full EXPERIVA cohort or to the broader population in the Peace River Valley may be limited.

In conclusion, our exploratory study lends some weight to the hypothesis that a predominant source contributes to Sr exposure in most exposed EXPERIVA participants. However, whether unconventional oil and gas exploitation contributes to it remains unclear. Our study has important limitations, and findings should be interpreted accordingly. Larger studies including isotope ratio measurements in biological, food/water, and environmental samples are needed to more precisely determine sources of Sr exposure and the extent of their contribution.

## Supplementary information

## Data Availability

The dataset analysed during the current study are not publicly available due to restrictions in our research agreements and ethical approval.
