# Supplementary material for: Probing sources of strontium exposure in pregnant individuals living near unconventional oil and gas wells using urinary 87Sr/86Sr isotope ratios
Source: J Expo Sci Environ Epidemiol. 2025 Jun 19;36(5):827–33. doi: 10.1038/s41370-025-00784-0 (PMC13538027; doi:10.1038/s41370-025-00784-0)
Supplement: Supplementary file 1 [file 41370_2025_784_MOESM1_ESM.pdf]

Supplementary information - Probing sources of strontium exposure in pregnant individuals living near unconventional oil and gas wells using urinary $^{87}Sr/^{86}Sr$ isotope ratios

Karel Houessionon[1,2], Bruna Saar de Almeida[3], David Widory[3], Michèle Bouchard[1,2,4], Vikki Ho[5,6], Coreen Daley[7], Élyse Caron-Beaudoin[7,8] Delphine Bosson-Rieutort[2,9], Marc-André Verner[1,2]

[1]Department of Occupational and Environmental Health, School of Public Health, Université de Montréal, Montréal, QC, Canada

[2]Centre de Recherche en Santé Publique, Université de Montréal and CIUSSS du Centre-Sud-de-l'Île-de-Montréal, Montreal, QC, Canada;

[3]GEOTOP, Université de Québec à Montréal, Montréal, QC, Canada

[4]Chaire d'analyse et de gestion des risques toxicologiques, Université de Montréal, Montréal, QC, Canada

[5]Department of Social and Preventive Medicine, School of Public Health, Université de Montréal, Montréal, QC, Canada

[6]Health Innovation and Evaluation Hub, Université de Montréal Hospital Research Centre (CRCHUM), Montréal, QC, Canada

[7]Department of Physical and Environmental Sciences, University of Toronto Scarborough, Toronto, ON, Canada

[8]Department of Health and Society, University of Toronto Scarborough, Toronto, ON, Canada

[9]Department of Health Policy, Management and Evaluation, School of Public Health, Université de Montréal, Montréal, QC, Canada

Corresponding author: Marc-André Verner (marc-andre.verner.1@umontreal.ca)

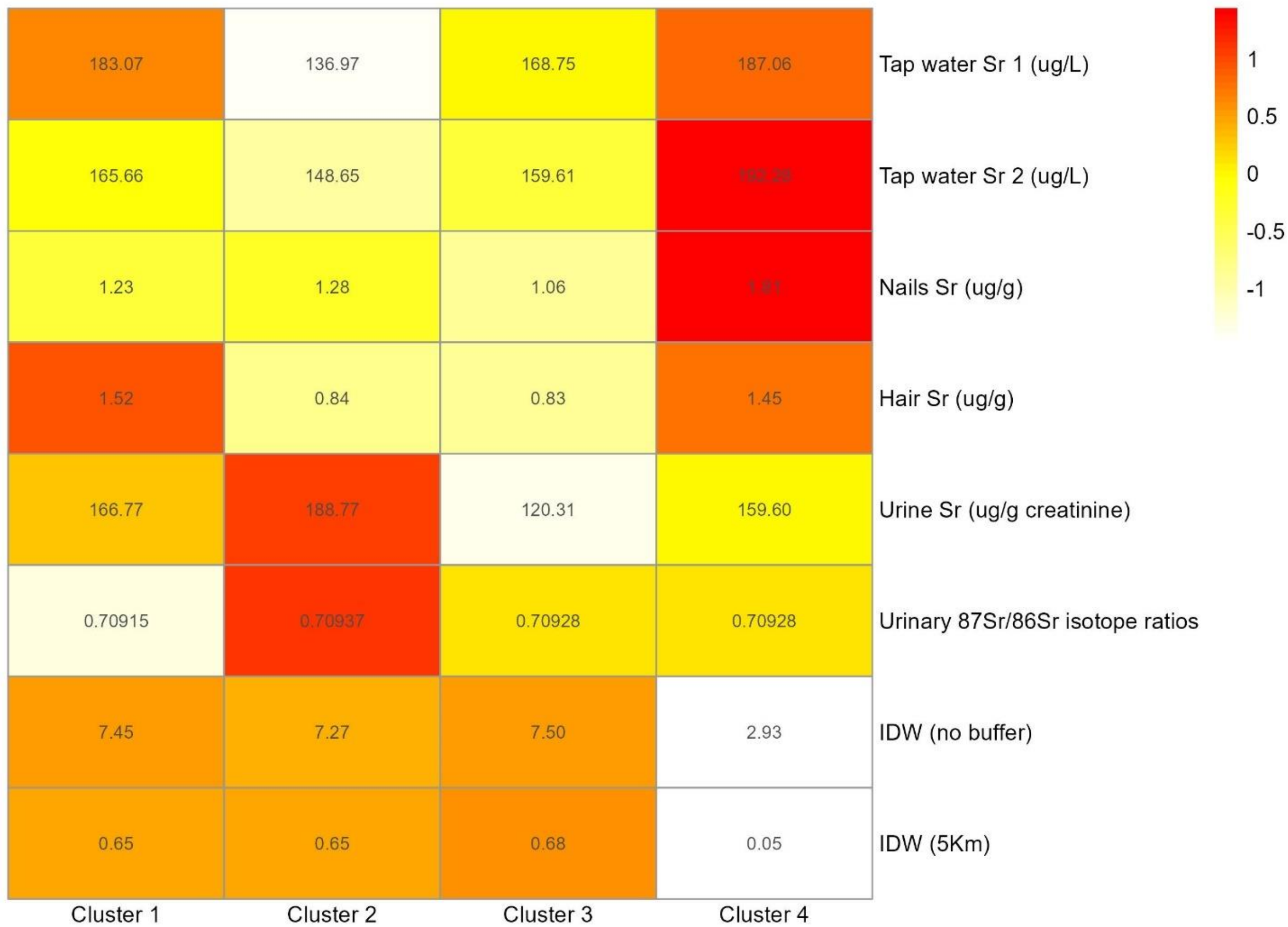

Figure S1. Heatmap of measured Sr concentrations (in hair, nails, urine and tap water) and unconventional oil and gas well density/proximity (IDW) metrics in the four clusters established through unsupervised clustering.

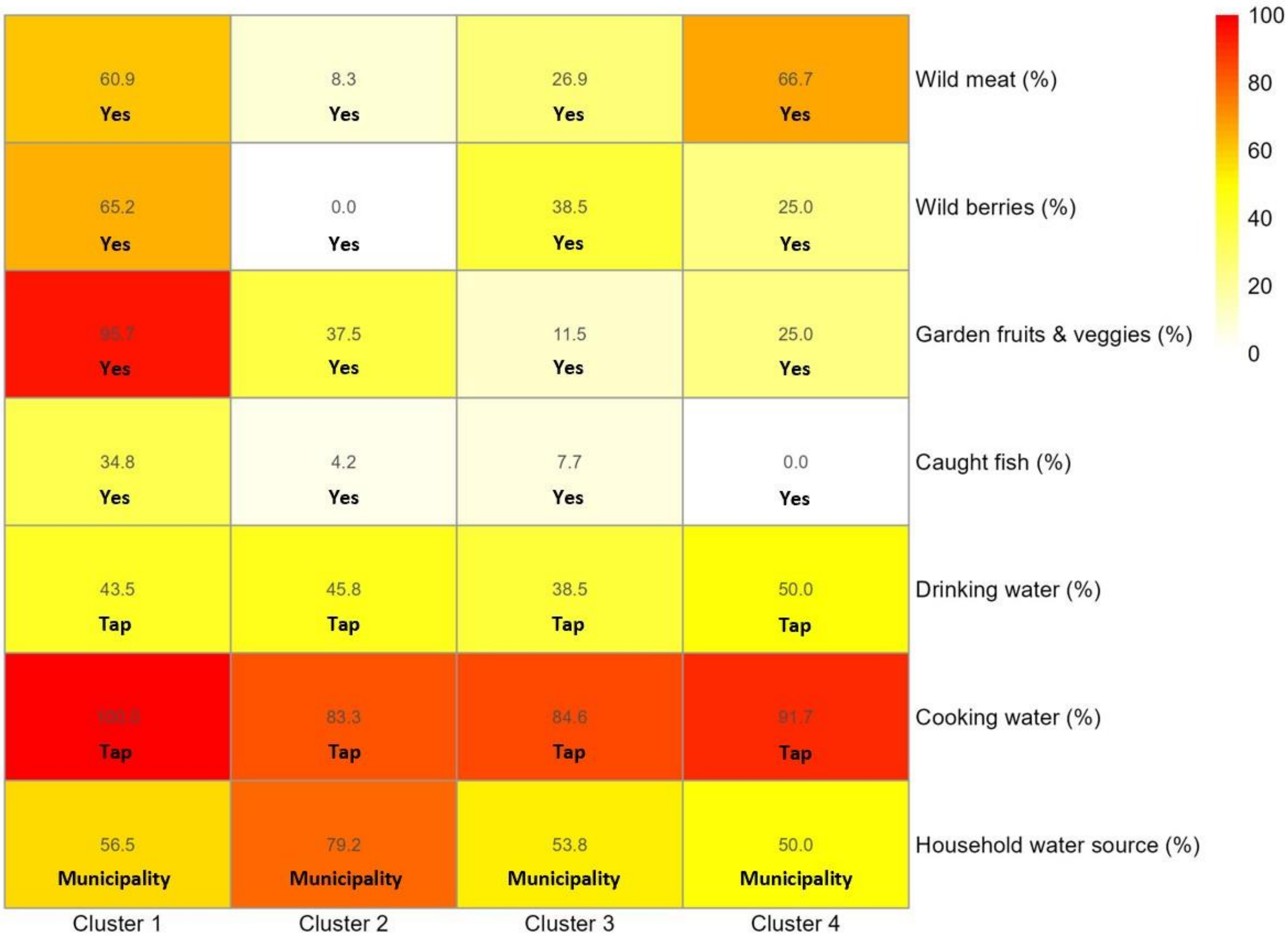

Figure. S2. Heatmap of socio-demographic and lifestyle characteristics in the four clusters established through unsupervised clustering.
